# Construction of Porous Organic/Inorganic Hybrid Polymers Based on Polyhedral Oligomeric Silsesquioxane for Energy Storage and Hydrogen Production from Water

**DOI:** 10.3390/polym15010182

**Published:** 2022-12-30

**Authors:** Mohamed Gamal Mohamed, Mohamed Hammad Elsayed, Yunsheng Ye, Maha Mohamed Samy, Ahmed E. Hassan, Tharwat Hassan Mansoure, Zhenhai Wen, Ho-Hsiu Chou, Kuei-Hsien Chen, Shiao-Wei Kuo

**Affiliations:** 1 Department of Materials and Optoelectronic Science, College of Semiconductor and Advanced Technology Research, Center for Functional Polymers and Supramolecular Materials, National Sun Yat-sen University, Kaohsiung 804, Taiwan; 2 Chemistry Department, Faculty of Science, Assiut University, Assiut 71515, Egypt; 3 Institute of Atomic and Molecular Sciences, Academia Sinica, Taipei 10617, Taiwan; 4 Department of Chemistry, Faculty of Science, Al-Azhar University, Nasr City, Cairo 11884, Egypt; 5 CAS Key Laboratory of Design and Assembly of Functional Nanostructures, Fujian Provincial Key Laboratory of Nanomaterials, Fujian Institute of Research on the Structure of Matter, Chinese Academy of Sciences, Fuzhou 350002, China; 6 University of Chinese Academy of Sciences, Beijing 100049, China; 7 Department of Chemical Engineering, National Tsing Hua University, Hsinchu 30013, Taiwan; 8 Department of Medicinal and Applied Chemistry, Kaohsiung Medical University, Kaohsiung 807, Taiwan

**Keywords:** porous organic/inorganic polymers, octavinylsilsesquioxane, triphenylamine, fluorene, supercapacitor, hydrogen production

## Abstract

In this study, we used effective and one-pot Heck coupling reactions under moderate reaction conditions to construct two new hybrid porous polymers (named OVS-P-TPA and OVS-P-F HPPs) with high yield, based on silsesquioxane cage nanoparticles through the reaction of octavinylsilsesquioxane (OVS) with different brominated pyrene (P-Br_4_), triphenylamine (TPA-Br_3_), and fluorene (F-Br_2_) as co-monomer units. The successful syntheses of both OVS-HPPs were tested using various instruments, such as X-ray photoelectron (XPS), solid-state ^13^C NMR, and Fourier transform infrared spectroscopy (FTIR) analyses. All spectroscopic data confirmed the successful incorporation and linkage of P, TPA, and F units into the POSS cage in order to form porous OVS-HPP materials. In addition, the thermogravimetric analysis (TGA) and N_2_ adsorption analyses revealed the thermal stabilities of OVS-P-F HPP (*T_d_*_10_ = 444 °C; char yield: 79 wt%), with a significant specific surface area of 375 m^2^ g^–1^ and a large pore volume of 0.69 cm^3^ g^–1^. According to electrochemical three-electrode performance, the OVS-P-F HPP precursor displayed superior capacitances of 292 F g^−1^ with a capacity retention of 99.8% compared to OVS-P-TPA HPP material. Interestingly, the OVS-P-TPA HPP showed a promising HER value of 701.9 µmol g^−1^ h^−1^, which is more than 12 times higher than that of OVS-P-F HPP (56.6 µmol g^−1^ h^−1^), based on photocatalytic experimental results.

## 1. Introduction

Supercapacitors (SCs) are regarded as an alternative technology to fuel cells, the usual capacitor, and batteries. They acquired widespread interest compared with other batteries and conventional capacitors due to their lower environmental impact, prolonged cycle life, and higher power capability [1,2,3,4,5,6,7,8]. SCs have two common types: electrode–electrolyte interfaces in electric double-layer capacitors (EDLCs), which are followed by physical adsorption; and pseudocapacitors, which involve faradaic interactions that occur between the electrolyte and organic moieties or active metal oxides [9,10,11,12,13,14,15,16]. As research advances, scientists are no longer concentrating only on developing the energy density of supercapacitors but are focusing more on improving the multifunctionality of supercapacitors, such as elastic wearable supercapacitors, smart energy storage windows, electrochromic supercapacitors (ESCs), self/charging supercapacitors, and so on [17,18,19,20,21,22]. Significantly, the performance of the supercapacitor is related to the electrode material which should fulfill the following advantages: higher electrical conductivity in order to facilitate high-rate capabilities and power densities, a large specific surface beside porosity, outstanding compatibility in order to ease ion diffusion and the ion/attainable surface area, and good distribution of pore size in order to achieve high specific capacitance and effective charge storage [23,24,25,26,27,28,29,30,31,32,33]. Hence, the most widespread materials that are utilized as the electrodes in SC applications are redox organic molecules, porous carbon materials, metal oxides, sulfides, hydroxides, and conjugated polymers (i.e., microporous polymers) because of their high surface area, versatility, adjustable porosity, obtainable raw materials, high electrical conductivity, high sustainability, and their remarkable stability under a large range of potential windows [34,35,36,37,38,39,40,41,42]. 

Porous organic polymers and porous organic hybrid polymers containing polyhedral oligomeric silsesquioxanes (POSS) have been immensely exercised in numerous applications, including hydrogen production, gas capture, chemical sensing catalysis, microelectronics, aerospace, iodine capture, biomedicine, water treatment, and energy storage [43,44,45,46,47,48]. In particular, cubic silsesquioxane molecules, which are considered an important kind of polyhedral oligomeric silsesquioxane, have attracted considerable attention in the scientific society, owing to their wonderful design from inorganic/organic hybrid materials, high surface area, tunable chemical properties, optical transparency, excellent mechanical toughness, and thermal stability [43,44,45,46,47,48,49,50,51,52]. The above molecules have a formula of (RSiO_1.5_) with a nanoscale diameter of about 1–3 nm which contain inorganic cages in their structures that provide superior advantages, as mentioned before [53,54,55,56,57]. Subsequently, various preparation methods have been reported in order to synthesize porous polymers using cubic silsesquioxane molecules as the building unit, such as the Heck coupling reaction which achieves the demand of preparing clean and low-price materials with high-performance capabilities for energy storage devices and other applications. For example, Ervithayasuporn’s group used a porous polymer nanocomposite based on OVS and porphyrin for capturing heavy metal anions and ions [58]. Zhang et al. prepared azo porous materials containing OVS as a cage and porphyrin for removing RhB and various metal ions from wastewater [59].

**Scheme 1:**
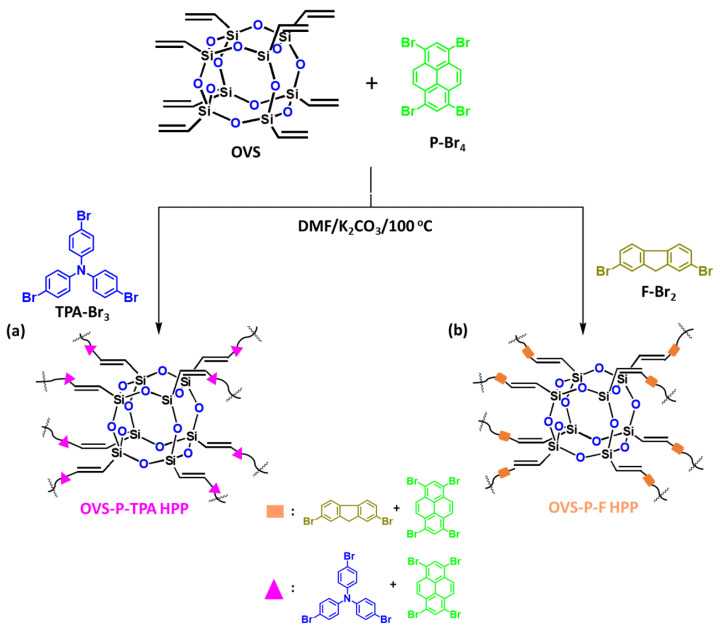
Preparation route of (**a**) OVS-P-TPA HPP and (**b**) OVS-P-F HPP through the Heck coupling reaction.

Motivated by these earlier reports, here, we have prepared two porous OVS-P-TPA and OVS-P-F HPPs with high yield using the Heck coupling reaction of the OVS cage with two different brominated comonomers (P/TPA and P/F) in the presence of Pd as a catalyst under moderate reaction conditions, as presented in Scheme 1. The molecular chemical structures, thermal stability (including temperature decomposition and carbon residues), crystallinities, surface morphologies, and porosity properties of the OVS-P-TPA and OVS-P-F HPPs were carefully investigated and discussed in detail using Brunauer–Emmett–Teller (BET) surface areas, X-ray photoelectron (XPS), and Fourier transforms infrared spectroscopy (FTIR) analyses. For the real application of these porous hybrid materials, two OVS-P-TPA and OVS-P-F HPPs were applied, and the electrochemical three-electrode performance and H_2_ evolution from water splitting were assessed. The data revealed that the POSS-P-F HPP precursor displayed high specific capacitances of 292 F g^−1^ with a capacity retention of 99.8% compared to the POSS-P-TPA HPP material. Interestingly, the OVS-P-TPA HPP shows a promising HER value of 701.9 µmol g^−1^ h^−1^ based on photocatalytic experimental results.

## 2. Materials and Methods

### 2.1. Materials

Octavinylsilsesquioxane (OVS), potassium carbonate (K_2_CO_3_), Pd(PPh_3_)_4_, iron(III) chloride (FeCl_3_), pyrene (P), bromine (Br_2_), N-bromosuccinimide (NBS), nitrobenzene, triphenylamine (TPA), and fluorene (F) were ordered from Alfa Aesar. All the organic solvents were obtained from Acros. 

### 2.2. Synthesis of P–Br_4_ [60,61]

Nitrobenzene (60 mL), Br_2_ (4 mL), and P molecules (2.00 g, 10 mmol) were introduced into a 100 mL two-necked flask that was filled with nitrogen. The mixture was then heated at 120 °C for 24 h, and the resulting P–Br_4_ was washed with EtOH before drying in a vacuum-sealed oven at 50 °C (4 g, 93%, Scheme 1). FTIR (Figure S1): 3053 (aromatic C–H stretching).

### 2.3. Synthesis of TPA-Br_3_ [39]

DMF (40 mL), NBS (3.00 g, 17.25 mmol), and TPA (1.38 g, 5.62 mmol) were combined in a 200 mL two-necked flask in an N_2_ atmosphere for 24 h at room temperature. Next, 300 mL of water and 200 mL of DCM were added to the reaction mixture. The DCM layer was dried over MgSO_4_ and evaporated in order to attain a white powder (2.83 g, 92%). M.p.: 142 °C (DSC, Figure S2). FTIR (Figure S3): 3078 (aromatic C–H stretching). ^1^H-NMR (Figure S4): 6.94–7.35 (aromatic protons).^13^C NMR (Figure S5): 146.80, 133.20, 126.20, and 116.40.

### 2.4. Synthesis of 2,7-Dibrmo-9H-Fluorene (F–Br_2_) [20]

F (4.00 g, 24.0 mmol), Br_2_ solution (1.29 mL, 25.27 mmol), CHCl_3_ (50 mL), and FeCl_3_ (0.134 g, 2.40 mmol) were injected into a 100 mL two-necked flask in an N_2_ atmosphere. The reaction was then held in the dark for 4 h. After this, CHCl_3_ was removed under a vacuum system in order to obtain F–Br_2_ as a white powder (7.80 g, 89%, Scheme S3). M.p.:165 °C (DSC, Figure S6). FTIR (Figure S7): 3056 (aromatic C–H stretching). ^1^H NMR (Figure S8): 3.90 (S, 2H) and 7.51–7. 66 (aromatic protons). ^13^C NMR (Figure S9): 145.36, 140.13, 130.98, 128.35, 122.06, and 36.69.

### 2.5. Synthesis of OVS-P-TPA HPP and OVS-P-F HPP 

A mixture of DMF (25 mL), Pd(PPh_3_)_4_ (0.032 g, 0.27 mmol), TPA-Br_3_ (1.45 g, 3 mmol) or F-Br_2_ (0.97, 3 mmol), OVS (0.5 g, 0.79 mmol), P-Br_4_ (0.04 g, 0.08 mmol), and K_2_CO_3_ (1.1 g, 7.9 mmol) was charged into a 50 mL two-necked round flask under an N_2_ atmosphere. The obtained solution was heated at 100 °C for 72 h. The solid was filtered and washed many times with water, THF, methanol, and acetone in order to obtain a red powder for OVS-P-TPA HPP (yield: 85%) and a yellow powder for OVS-P-F HPP (yield: 80%). The schematic route of OVS-P-TPA HPP and OVS-P-F HPP is displayed in Scheme 1a,b, respectively. 

### 2.6. Photocatalytic H_2_ Evolution Experiment 

In a typical measurement, 5 mg of OVS-P-TPA HPP or OVS-O-F HPP photocatalyst was dispersed by sonication in a 10 mL solution (water/MeOH (2/1)) containing 0.1 M ascorbic acid (AA), which was then inserted into the reaction glass container and sealed tightly with a rubber septum. Prior to illumination, the aerobic oxygen was removed from the sample by evacuating the solution mixture and then exposing it to a continuous stream of Ar gas for 30 min. The catalyst solution was exposed to light from a 350 W Xe lamp (1000 W m^−2^; λ > 380 nm) that had passed through a 380 nm band-pass filter. Using a gas-tight syringe, the hydrogen sample was taken every hour and injected in a Shimazhu GC-2014 gas chromatograph, using Ar as the carrier gas. Hydrogen was detected with a thermal conductivity detector, referring to the standard hydrogen gases with known concentrations [62,63].

### 2.7. Computational Details

The density functional theory (DFT) of the DMol3 code [64] was used in order to obtain the optimized structure and electronic properties. The generalized gradient approximation of the Perdew–Burke–Ernzerhof functional (GGA + PBE) was adopted throughout all the calculations [65]. The system’s energy was converged to 10^−5^ Ha, and the max force and the max displacements were 0.002 Ha/Å and 0.005 Å, respectively. In order to ensure self-consistent field (SCF) convergence accuracy, a self-consistent field (SCF) was converged to 10^−6^ Ha. In addition to their electron densities, the energy levels of the highest occupied molecular orbital (HOMO) and the lowest unoccupied molecular orbital (LUMO) were computed [66].

## 3. Results

### 3.1. Synthesis and Characterization of OVS-P-TPA and OVS-P-F HPPs

**Figure 1:**
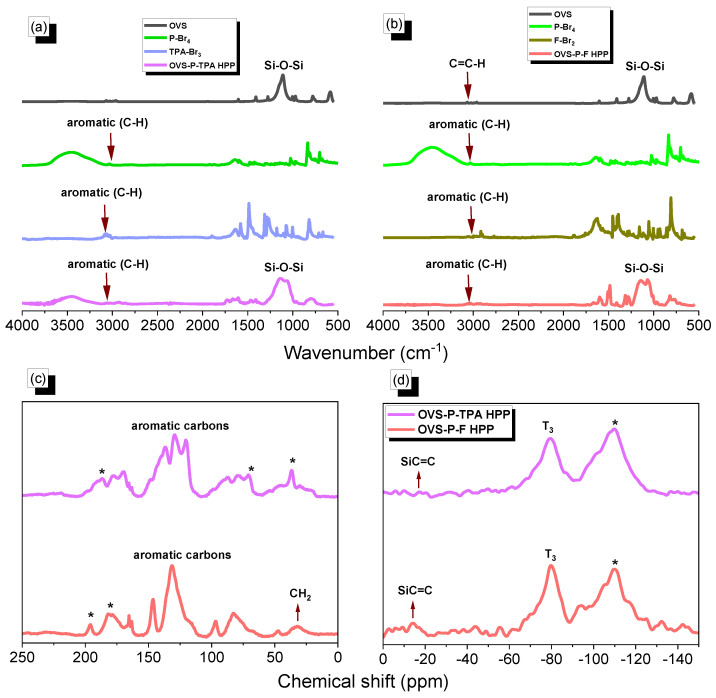
FTIR analyses of (**a**) OVS, P-Br_4_, TPA-Br_3_, and OVS-P-TPA HPP; (**b**) OVS, P-Br_4_, F-Br_2_, and OVS-P-F HPP; (**c**) the ^13^C NMR solid-state profile; and (**d**) the ^29^Si NMR solid-state profile of OVS-P-TPA HPP and OVS-P-F HPP. * denotes the side band of solid-state nuclear magnetic resonance spectroscopy (NMR).

The preparation route of OVS-HPPs (OVS-P-TPA and OVS-P-F HPPs) is presented in Scheme 1. First, the P-Br_4_ monomer was synthesized using the bromination reaction of P with Br_2_, using nitrobenzene as a solvent, as shown in Scheme S1. Secondly, TPA-Br_3_ was easily synthesized through the reaction of TPA with Br_2_ solution in DMF at 25 °C (see Scheme S2). Finally, F-Br_2_ was prepared with a high yield through the reaction of the F molecule with Br_2_ in CHCl_3_ (see Scheme S3). The NMR and FTIR data of the P-Br_4_, TPA-Br_3,_ and F-Br_2_ monomers in this study confirmed their successful synthesis [see the experimental part of this study]. Scheme 1 displays the synthesis route for the synthesis of our two new porous hybrid polymers (OVS-HPPs) based on the OVS unit (named OVS-P-TPA and OVS-P-F HPPs) via a simple and efficient Heck coupling reaction in a mixed solution containing DMF, K_2_CO_3_, and Pd as a catalyst. The mole ratios between all the monomers (OVS/TPA-Br_3_ or F-Br_2_/P-Br_4_), which were used in the preparation of the OVS-HPPs, was 1:3.8:0.1 [67]. We used this ratio to get a high surface area of these HPP materials and to increase the amount of pyrene moiety content as a donor unit in the HPP framework in order to adjust the energy band gap and investigate their potential applications in energy storage and hydrogen evolution. The OVS-P-TPA HPP was obtained through the reaction of OVS with P-Br_4_ and TPA-Br_3_. This reaction produced OVS-P-TPA as a red powder [Scheme 1a]. OVS-P-F HPP was obtained through the reaction of OVS with P-Br_4_ and F-Br_2_, which produced a yellow solid [Scheme 1b]. After completing the Heck coupling reaction, we revealed that the obtained OVS-HPP materials were not soluble in MeOH, H_2_O, DMF, DCM, THF, and acetone, indicating that our porous OVS-HPPs had a high crosslinking density and a high degree of polymerization. The matching bands for stretching Si-O-Si, C=C, and C=C-H groups in the OVS compound showed at 1107, 1600, and 3066 cm^−1^, respectively, as shown in Figure 1a. According to Figure 1a,b, Figures S10 and S11, the band of the C-H aromatic in P-Br_4_, TPA-Br_3_, and F-Br_2_ are represented at 3053, 3078, and 3056 cm^−1^, respectively. Figure 1a,b, Figures S10 and S11 show that the peaks for aliphatic C-H and C=C units in the FTIR profiles of the OVS-P-TPA and OVS-P-F HPPs were found in the ranges of 2981–2964 and 1600–1590 cm^−1^, respectively. As anticipated, the Si-O-Si absorption bands in both the OVS-P-TPA and the OVS-P-F HPPs (Figure 1a,b) were wider compared to the Si-O-Si unit in the OVS compound (Figure 1). This was attributed to the development of cross-linked networks. Figure 1c reveals that the signal for aromatic carbons appeared in the range of 142–117 ppm for OVS-P-TPA HPP and 146–114 ppm for OVS-P-F HPP. The signal of the carbon nuclei of the C-N unit appeared at 146 ppm for OVS-P-TPA HPP. In addition, in the ^13^C NMR solid-state profile, OVS-P-F HPP had a signal located at 36.6 ppm, corresponding to the CH_2_ group. Moreover, the presence of OVS cage units in all the OVS-HPP materials was proven using ^29^Si NMR solid-state profile (Figure 1d). The two OVS-HPP samples displayed two signals centered near −14 and −80 ppm due to the Si-C=C and T_3_, respectively.

**Figure 2:**
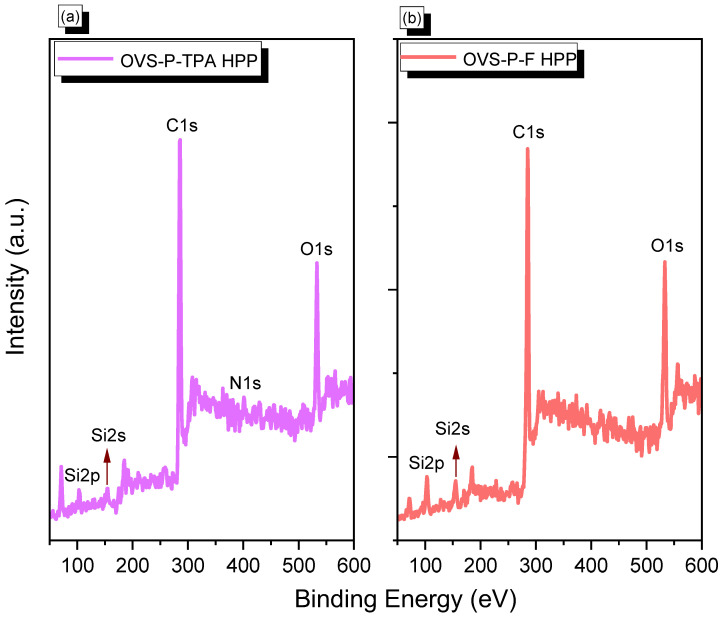
XPS analysis of (**a**) OVS-P-TPA HPP and (**b**) OVS-P-F HPP.

The surface chemical composition of OVS-P-TPA HPP and OVS-P-F HPP were detected in X-ray photoelectron spectroscopic (XPS) measurements. As can be seen in Figure 2, the Si2p (103.13 eV), Si2s (153.31 eV), C1s (285.80 eV), and N1s (401.12 eV) peaks appeared in the OVS-P-TPA HPP precursor (Figure 2a), whereas the Si2p (103.48 eV), Si2s (153.84 eV), and C1s (285.34 eV) signals appeared in the OVS-P-F HPP framework (Figure 2b) [68]. All the analyses above, including FTIR, XPS, ^13^C, and ^29^Si NMR solid-state analyses, strongly demonstrated the successful synthesis of OVS-P-TPA HPP and OVS-P-F HPP.

**Figure 3:**
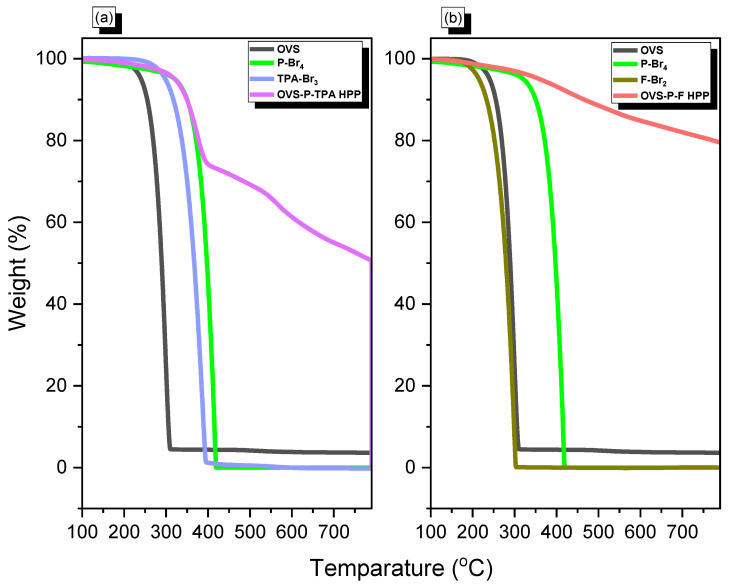
TGA analyses of (**a**) OVS, P-Br_4_, TPA-Br_3_, and OVS-P-TPA HPP and (**b**) OVS, P-Br_4_, F-Br_2_, and OVS-P-F HPP under N_2_.

Figure 3 displays the thermogravimetric analysis (TGA) profiles of OVS-P-TPA HPP and OVS-P-F HPP and their corresponding building blocks under an N_2_ atmosphere at 800 °C. The OVS-P-F HPP showed thermal decomposition temperatures of *T_d_*_5_, *T_d_*_10_ with a char yield at 228, 283°C, and 51 wt%, respectively. For OVS-P-TPA HPP and OVS-P-F HPP; these were 349, 444 °C and 79 wt%, respectively. Interestingly, after the Heck polymerization reaction, both OVS-HPPs possessed outstanding thermal stability properties compared with their building blocks, as shown in Figure 3a,b. The thermal stability results (including *T_d_*_5_, *T_d_*_10,_ and the char yields) of the OVS-HPPs and all the synthesized monomers used in this study are summarized in Table 1 and Table S1. The X-ray patterns (Figure S12) displayed amorphous characteristics for the OVS-HPPs materials. In addition, a gradual high X-ray diffraction peak can be seen at 2θ = 22° for the presence of Si-O-Si groups in the OVS cage [67].

**Figure 4:**
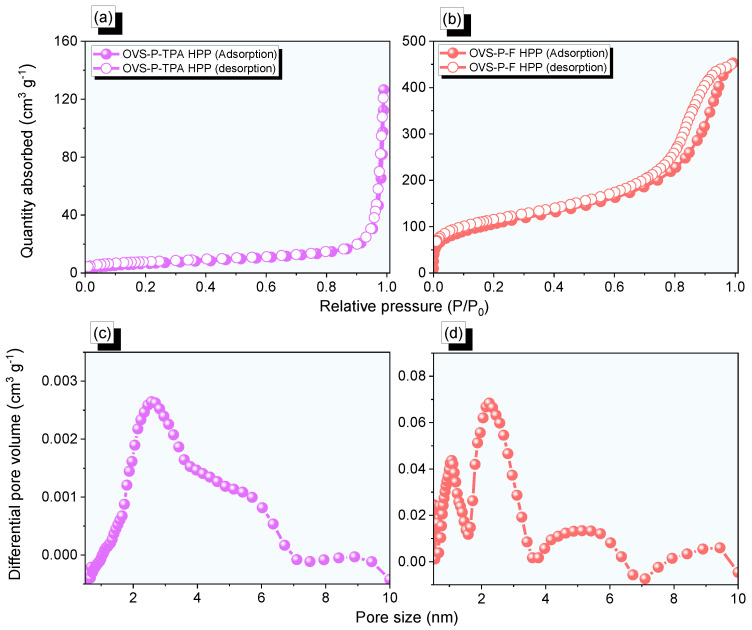
BET and pore size profiles of (**a**,**c**) OVS-P-TPA HPP and (**b**,**d**) OVS-P-F HPP.

In order to confirm the porosity properties of the OVS-HPPs, the BET analyses were performed at 77 K and 1 bar (Figure 4). According to the IUPAC classification, the N_2_ isotherm profile for the OVS-P-TPA HPP sample (Figure 4a) was type III, and for the OVS-P-F HPP framework was the type I and IV (Figure 4b). OVS-P-TPA HPP contains mainly meso- and macro-pores, and OVS-P-F HPP shows a mix of micro- and mesopores at high relative pressures (P/P0 > 0.9), based on BET analyses. The specific surface area and total pore volumes of the OVS-P-TPA and OVS-P-F HPPs were 60 m^2^ g ^−1^ and 0.19 cm^3^, and 375 m^2^ g ^−1^ and 0.69 cm^3^ g ^−1^, respectively (Table 1). We used the nonlocal density functional theory method to calculate the pore size distribution for the OVS-HPPs materials (Figure 4c,d), and the pore diameters were found to be 2.56 and 2.20 nm, respectively, for the OVS-P-TPA and OVS-P-F HPPs. In addition, the relative content of micropores for the OVS-P-TPA and OVS-P-F HPPs was 0.12 and 0.21, respectively.

**Figure 5:**
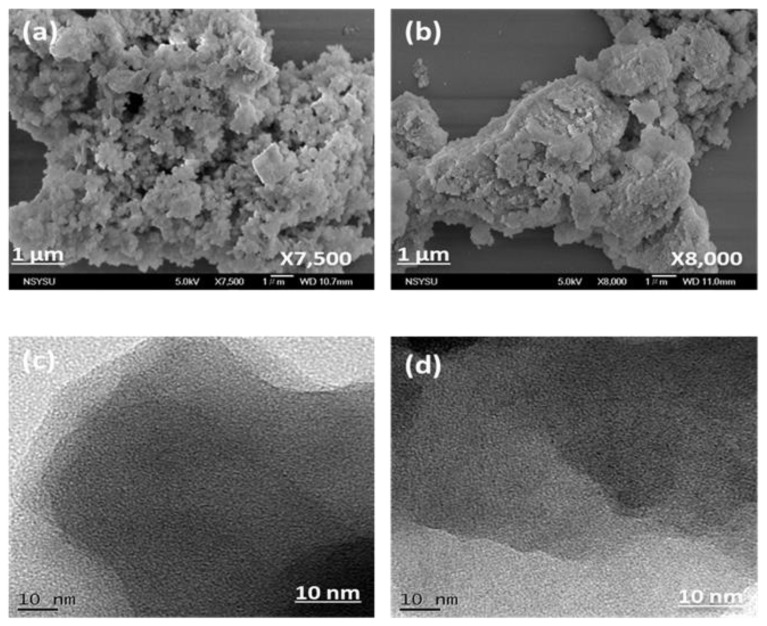
SEM and TEM images of (**a**,**c**) OVS-P-TPA HPP and (**b**,**d**) OVS-P-F HPP.

The shape and porosity parameters of the OVS-HPPs were evaluated using SEM and TEM measurements (Figure 5). The OVS-P-TPA and OVS-P-F HPPs featured irregular aggregated sphere structures and aggregated irregular cloud-like structures, respectively, according to the SEM images (Figure 5a,b). Moreover, the TEM images of the OVS-HPP materials are shown in Figure 5c,d; the images revealed the presence of pinholes and the presence of dark and bright regions in their structures.

### 3.2. Electrochemical Analysis of the OVS-P-TPA and OVS-P-F HPPs 

**Figure 6:**
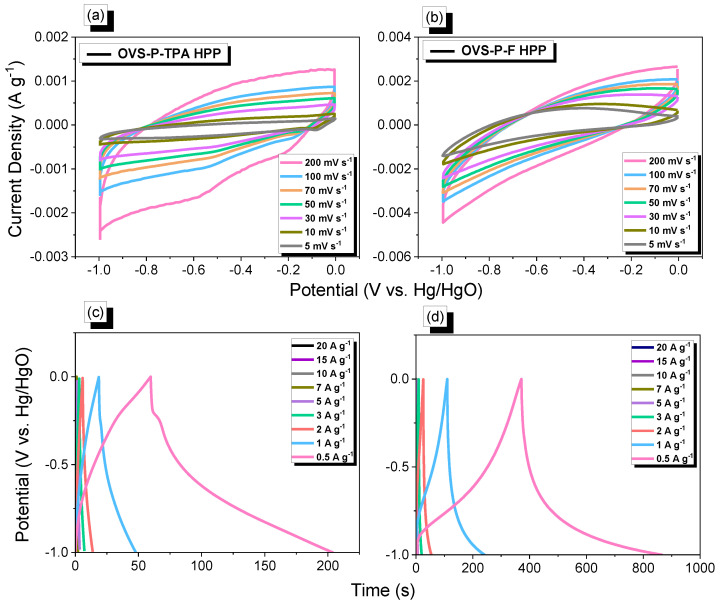
CV and GCD curves of (**a**,**c**) OVS-P-TPA HPP and (**b**,**d**) OVS-P-F HPP.

A three-electrode setup was used to examine the electrochemical performance of the OVS-HPP materials (OVS-P-TPA and OVS-P-F HPPs) using cyclic voltammetry (CV) and galvanostatic charge–discharge (GCD) measurements. Figure 6a,b shows the CV curves of the two synthesized OVS-HPP frameworks and the configuration of the electrochemical cell used for the measurements. The voltage windows ranged from 0.00 to 1.00 V (vs. Hg/HgO) at a sweep speed ranging from 5 to 200 mV s^−1^. Furthermore, when recorded at the highest scan rate of 200 mV s^−1^, the CV curves of the OVS-HPP samples showed rectangle-shaped shapes with the appearance of humps, indicating that this capacitive response was primarily caused by EDLC with a small amount of pseudocapacitance [2,12,35,69]. The integrated area of the OVS-P-F HPP sample was higher than that of the OVS-P-TPA HPP sample, revealing its superior electrochemical performance. This superior electrochemical performance of the OVS-P-F HPP sample was due to its high porosity properties [12]. Figure 6c,d presents the GCD curves of the OVS-P-TPA and OVS-P-F HPPs. The GCD curves of the OVS-HPP samples revealed triangular-like shapes with a slight bend at different current densities (0.5–20 A g^−1^), indicating the presence of pseudocapacitance and EDLC characteristics [2,12,35,69].

**Figure 7:**
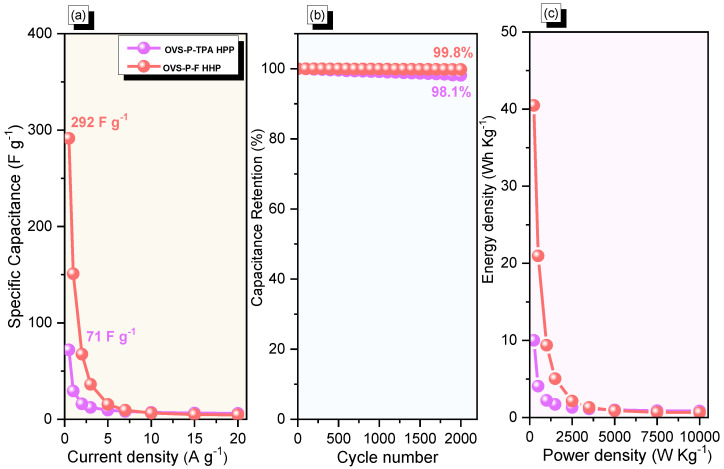
(**a**) Specific capacitance, (**b**) durability, and (**c**) Ragone plots of OVS-P-TPA HPP and OVS-P-F HPP.

Figure 7a presents the capacitances of the OVS-HPPs determined using Equation (S1) (from the GCD curves). The OVS-P-F HPP sample displayed excellent capacitance (292 F g^–1^) among the tested OVS-P-TPA HPPs (72 F g^–1^) at 0.5 A g^–1^. This behavior could be explained in terms of its higher surface area (375 m^2^ g^−1^), and the existence of phenyl rings with abundant electrons, which make electrolytes more easily accessible to the surface of the electrode [2,12,35,69]. In addition, the capacitances of the OVS-HPP-based samples reduced as the current density increased from 0.5 to 20 A g^−1^ due to inadequate available time for ion diffusion and adsorption inside the tiniest pores inside a big particle at high current densities [3,12,69]. Additionally, compared to the other porous materials, the OVS-P-F HPP-based material showed good capacitance (Table S2) [12,35,69,70,71]. GCD measurements were used to examine the stability of the OVS-HPP samples after 2000 cycles at 10 A g^−1^ (Figure 7b). The OVS-P-TPA and OVS-P-F HPP samples displayed good cycling stability (Figure 7b), with 98.1 and 99.8% retention, respectively. The Ragone plot (Figure 7c) showed that the energy densities of the OVS-P-TPA and OVS-P-F HPP samples were 10.02 and 40.5 Wh Kg^−1^, respectively.

### 3.3. Photocatalytic Performance of OVS-P-TPA HPP and OVS-P-F HPP for H_2_ Production

**Figure 8:**
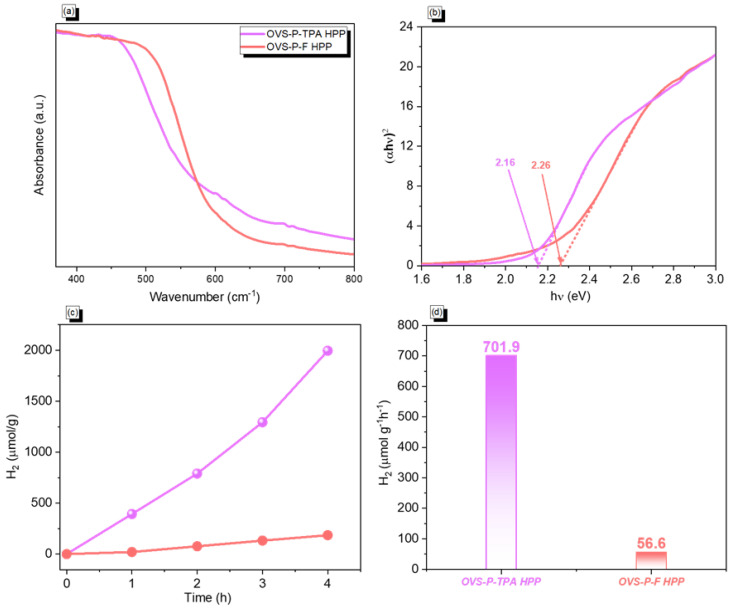
(**a**) UV-Vis DRS absorption spectra of the OVS-HPPs, (**b**) the Tauc plot of (αhν)^2^ versus (hν) from the UV-Vis spectra for calculating the bandgap of the OVS-HPPs, (**c**) time course of the produced H_2_, and (**d**) HER of the two polymers. Conditions: ascorbic acid (AA, 0.1 M) and a xenon lamp light source (AM1.5, λ > 380 nm, and 1000 W m^−2^).

The ultraviolet-visible diffuse reflectance spectroscopy (UV-Vis DRS) was used to investigate the light absorption properties of the polymers, as shown in Figure 8a. The two polymers demonstrated good absorption in the visible light region, indicating their ability to harvest large amounts of visible light during the photocatalytic reaction. The onset of the absorption spectrum of the two polymers approximately reached 600 and 650 nm for OVS-P-F HPP and OVS-P-TPA HPP, respectively. The bandgap (E_g_) of the polymers was determined from the Tauc plot of (αhν)^2^ versus (hν) from the UV-Vis spectra and by extrapolation of the linear part of the curve to the energy axis in order to obtain the optical bandgap based on the equation: αhν = A(hν − E_g_)^γ^, where α is the absorption coefficient, A is an energy-independent constant, E_g_ is the optical band gap, h is the plank constant, v is the velocity, and γ is the electronic transition. As shown in Figure 8b, the E_g_ values of the OVS-P-F HPP and OVS-P-TPA HPP polymers were calculated to be 2.26 and 2.16 eV, respectively. Next, we tested the polymers as photocatalysts for light-driven hydrogen evolution. In the presence of AA as a sacrificial reagent and without a Pt co-catalyst, we recorded the hydrogen evolution kinetic curve to explore the H_2_ evolution efficiency of the polymer photocatalyst, as shown in Figure 8c. The hydrogen evolution rate (HER) was obtained from the kinetic curves (Figure 8d). The total amount of H_2_ produced by the OVS-P-TPA HPP and OVS-P-F HPP polymer photocatalysts reached 2000 and 185 µmol/g after 4 h of the reaction, respectively (Figure 8c). The HER was calculated for the two polymers from the kinetic curve presented in Figure 8d. The OVS-P-TPA HPP sample shows a promising HER value of 701.9 µmol g^−1^ h^−1^, which is more than 12 times higher than that of OVS-P-F HPP (56.6 µmol g^−1^ h^−1^). The high HER value of OVS-P-TPA HPP is due to the donor–acceptor structure between the pyrene and triphenylamine moieties of the OVS-P-TPA HPP polymer, leading to separation of the charge carrier and then the enhancement of photocatalytic activity.

**Figure 9:**
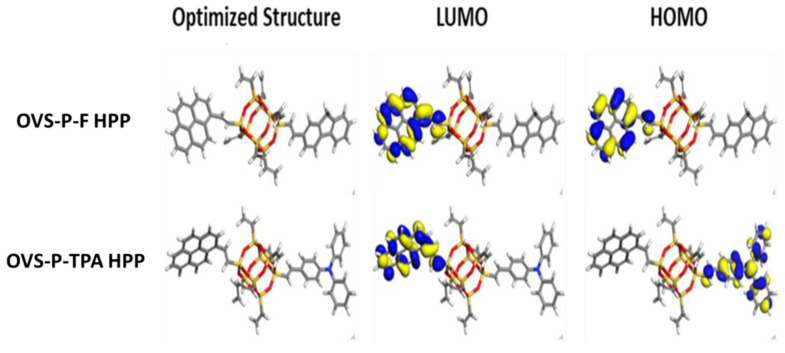
Optimized geometries, the density of HOMO, and the density of LUMO frontier molecular orbitals of the OVS-HPPs.

To determine how the donor and acceptor fragments influenced the photoelectric properties of the examined compounds, we calculated the charge density of the high occupied molecular orbital (HOMO) and low unoccupied molecular orbital (LUMO) energy levels using the density functional theory (DFT) of the DMol3 code. Figure 9 shows that highly pronounced spatial separation of the HOMOs and LUMOs can be observed for OVS-P-TPA HPP in contrast to OVS-P-F HPP. The LUMO orbital in the OVS-P-TPA HPP sample was mainly localized over the pyrene donor, whereas the HOMO orbital was localized over the triphenyl acceptor. On the other hand, in the case of the OVS-P-F HPP polymer, the HOMO and LUMO orbitals were localized over the pyrene moiety, and no charge separation was observed. Thus, we expect that OVS-P-TPA HPP has a better ability to separate the photogenerated e$#x2212;/h+ pairs and higher photocatalytic efficiency than other molecules. This distribution indicates high electron delocalization, and significant charge transfer (CT) occurs inside the investigated molecule under light irradiation.

## 4. Conclusions

In this study, we successfully constructed and designed OVSP-TPA HPP and POSS-P-F HPP using simple Heck coupling under moderate and suitable reaction conditions through the reaction of an OVS inorganic cage with brominated P/TPA or P/F as comonomers. All spectroscopic results revealed the successful syntheses of both OVS-HPPs with good yield. The POSS-P-F HPP sample had a *T_d_*_10_ of 444 °C, with a char yield of 79 wt%, as well as a high specific surface area of 375 m^2^ g^–1^ and a pore volume of 0.69 cm^3^ g^–1^. According to electrochemical three-electrode performance, the POSS-P-TPA and POSS-P-F HPPs precursor capacitances were 72 and 292 F g^−1^, respectively. Furthermore, the OVS-P-TPA HPP had a promising HER value of 701.9 µmol g^−1^ h^−1^ due to the donor–acceptor structure that was obtained between the P and TPA moieties of the OVS-P-TPA HPP.

## Figures and Tables

**Table 1:** TGA, BET, and capacitance data for the OVS-HPPs materials.

Sample	*T_d_*_10_ (°C)	Char Yield (wt.%)	Surface Area (m^2^ g^–1^)	Pore Volume (cm^3^ g^–1^)	Pore Size (nm)	Capacity at 0.5 A g^–1^ (F g^–1^)
OVS-P-TPA HPP	283	51	60	0.19	2.56	71
OVS-P-F HPP	444	79	375	0.69	2.2	296

## Data Availability

The data presented in this study are available on request from the corresponding author.

## Supplementary Materials

The following supporting information can be downloaded at: https://www.mdpi.com/article/10.3390/polym15010182/s1, Scheme S1: Synthesis of P-Br_4_ from pyrene in the presence of Br_2_; Scheme S2: Synthesis of TPA-Br_3_ from TPA in the presence of NBS and DMF as a solvent; Scheme S3: Synthesis of F-Br_2_ from fluorene in the presence of Br_2_; Figure S1: FTIR spectrum of P-Br_4_; Figure S2: DSC profile of TPA-Br_3_; Figure S3: FTIR spectrum of TPA-Br_3_; Figure S4: ^1^H NMR spectrum of TPA-Br_3_; Figure S5: ^13^C NMR spectrum of TPA-Br_3_; Figure S6: DSC profile of F-Br_2_; Figure S7: FTIR spectrum of F-Br_2_; Figure S8: ^1^H NMR spectrum of F-Br_2_; Figure S9: ^13^C NMR spectrum of F-Br_2_; Figure S10: FTIR analyses of OVS, P-Br_4_, TPA-Br_3_, and OVS-P-TPA HPP; Figure S11: FTIR analyses of OVS, P-Br_4_, F-Br_2_, and OVS-P-F HPP; Figure S12: XRD patterns of OVS-P-TPA and OVS-P-F HPPs; Table S1: TGA data of OVS-HPPs and synthesized monomers in this study; Table S2: Summarizes the specific capacitances of POSS-P-TPA HPP and POSS-P-F HPP compared to other organic and inorganic porous materials.
