# Supplementary material for: Construction of Porous Organic/Inorganic Hybrid Polymers Based on Polyhedral Oligomeric Silsesquioxane for Energy Storage and Hydrogen Production from Water
Source: Polymers (Basel). 2022 Dec 30;15(1):182. doi: 10.3390/polym15010182 (PMC9824186; doi:10.3390/polym15010182)
Supplement: Supplementary file 1 [file polymers-15-00182-s001.zip › polymers-2025222-supplementary.pdf]

Supplementary Materials

Characterization

FTIR spectra were collected on a Bruker Tensor 27 FTIR spectrophotometer with a resolution of 4 cm^{-1} by using KBr disk method. ^{13}C nuclear magnetic resonance (NMR) spectra was examined by using an INOVA 500 instrument with DMSO as the solvent and TMS as the external standard. Chemical shifts are reported in parts per million (ppm). The curing behavior and thermal stabilities of the samples were performed by using a TG Q-50 thermogravimetric analyzer under a N_2 atmosphere; the cured sample (ca. 5 mg) was put in a Pt cell with heating rate of 20 °C min^{-1} from 100 to 800 °C under a N_2 flow rate of 60 mL min^{-1}. Wide-angle X-ray diffraction (WAXD) patterns were measured by the wiggler beamline BL17A1 of the National Synchrotron Radiation Research Center (NSRRC), Taiwan. A triangular bent Si (111) single crystal was used to get a monochromated beam having a wavelength (λ) of 1.33 Å. The morphologies of the polymer samples were examined by Field emission scanning electron microscopy (FE-SEM; JEOL JSM7610F) and also by transmission electron microscope (TEM) using JEOL-2100 instrument at an accelerating voltage of 200 kV. BET surface area and porosimetry measurements of samples (ca. 40–100 mg) were measured using BEL Master™/BEL sim™ (v. 3.0.0). N_2 adsorption and desorption isotherms were generated through incremental exposure to ultrahigh-purity N_2 (up to ca. 1 atm) in a liquid N_2 (77 K) bath. Surface parameters were calculated using BET adsorption models in the instrument's software. The pore size of the prepared samples was determined by using nonlocal density functional theory (NLDFT).

Electrochemical Analysis

Working Electrode Cleaning: Prior to use, the glassy carbon electrode (GCE) was polished several times with 0.05-μm alumina powder, washed with EtOH after each polishing step, cleaned through sonication (5 min) in a water bath, washed with EtOH, and then dried in air.

Electrochemical Characterization: The electrochemical experiments were performed in a three-electrode cell using an Autolab potentiostat (PGSTAT204) and 1 M KOH as the aqueous electrolyte. The GCE was used as the working electrode (diameter: 5.61 mm; 0.2475 cm^2); a Pt wire was used as the counter electrode; Hg/HgO (RE-1B, BAS) was the reference electrode. All reported potentials refer to the Hg/HgO potential. A slurry was prepared by dispersing POSS-P-TPA HPP or POSS-P-F HPP (45 wt %), carbon black (45 wt %), and Nafion (10 wt %) in a mixture of (EtOH/ H_2O) (200 μL: 800 μL) and then sonicating for 1 h. A portion of this slurry (10 μL) was pipetted onto the tip of the electrode, which was then dried in air for 30 min prior to use. The electrochemical performance was studied through CV at various sweep rates (5–200 mV s^{-1}) and through the GCD method in the potential range from −1.0 V and 0.0 V (vs. Hg/HgO) at various current densities (0.5–20 A g^{-1}) in 1 M KOH as the aqueous electrolyte solution.

The specific capacitance was calculated from the GCD data using the equation.

$$C_s = (I\Delta t)/(m\Delta V) \quad \text{(S1)}$$

Where C_s (F g^{-1}) is the specific capacitance of the supercapacitor, I (A) is the discharge current, ΔV (V) is the potential window, Δt (s) is the discharge time, and m (g) is the mass of the NPC on the electrode. The energy density (E, W h kg^{-1}) and power density (P, W kg^{-1}) were calculated using the equations.

$$E = 1000C(\Delta V)^2/(2 \times 3600) \quad \text{(S2)}$$

$$P = E/(t/3600) \quad \text{(S3)}$$

Table S1. TGA data of OVS-HPPs and synthesized monomers in this study.

Sample	T_{d10} (°C)	Char Yield (wt.%)

OVS-P-TPA HPP	283	51
OVS-P-F HPP	444	79
OVS	255	3.6
P-Br_4	350	0
TPA-Br_3	317	0
F-Br_2	233	0

Table S2. Summarizes the specific capacitances of POSS-P-TPA HPP and POSS-P-F HPP compared to others organic and inorganic porous materials.

Electrode	S_{BET} ($m^2\ g^{-1}$)	Capacitance	Ref.
POSS-P-TPA HPP	364	72 F g^{-1} at 0.5 A g^{-1}	This work
POSS-P-F HPP	611	292 F g^{-1} at 0.5 A g^{-1}	This work
POSS-A-POIP	426	152.5 F g^{-1} at 0.5 A g^{-1}	[12]
POSS-F-POIP	452	36.2 F g^{-1} at 0.5 A g^{-1}	[12]
TBN-Py-CMP	473	31 F g–1 at 0.5 A g^{-1}	[70]
TBN-TPE-CMP	1150	18.45 F g^{-1} at 0.5 A g^{-1}	[70]
TBN-Car-CMP	762	18.90 F g^{-1} at 0.5 A g^{-1}	[70]
TPE-FFC-CMP	8	4.8 F g^{-1} at 0.5 A g^{-1}	[35]
Py-FFC-CMP	50	5.07 F g^{-1} at 0.5 A g^{-1}	[35]
C1-CMP-1	608	31 F g^{-1} at 20 mV s^{-1}	[71]
C2-CMP-2	577	66.1 F g^{-1} at 20 mV s^{-1}	[71]
N1-CMP-1	791	96.5 F g^{-1} at 20 mV s^{-1}	[71]
CoPc-CMP	-	13.8 F g^{-1} at 1.0 A g^{-1}	[69]

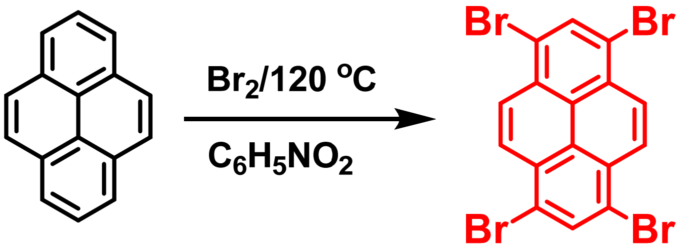

Scheme S1. Synthesis of P-Br_4 from pyrene in the presence of Br_2.

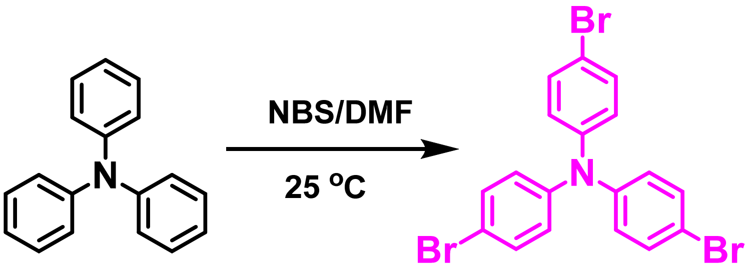

Scheme S2. Synthesis of TPA-Br_3 from TPA in the presence of NBS and DMF as a solvent.

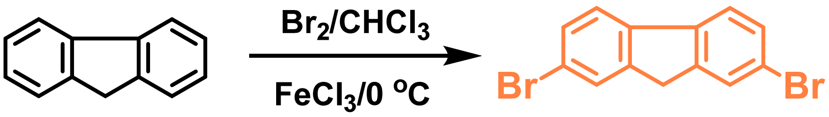

Scheme S3. Synthesis of F-Br_2 from fluorene in the presence of Br_2.

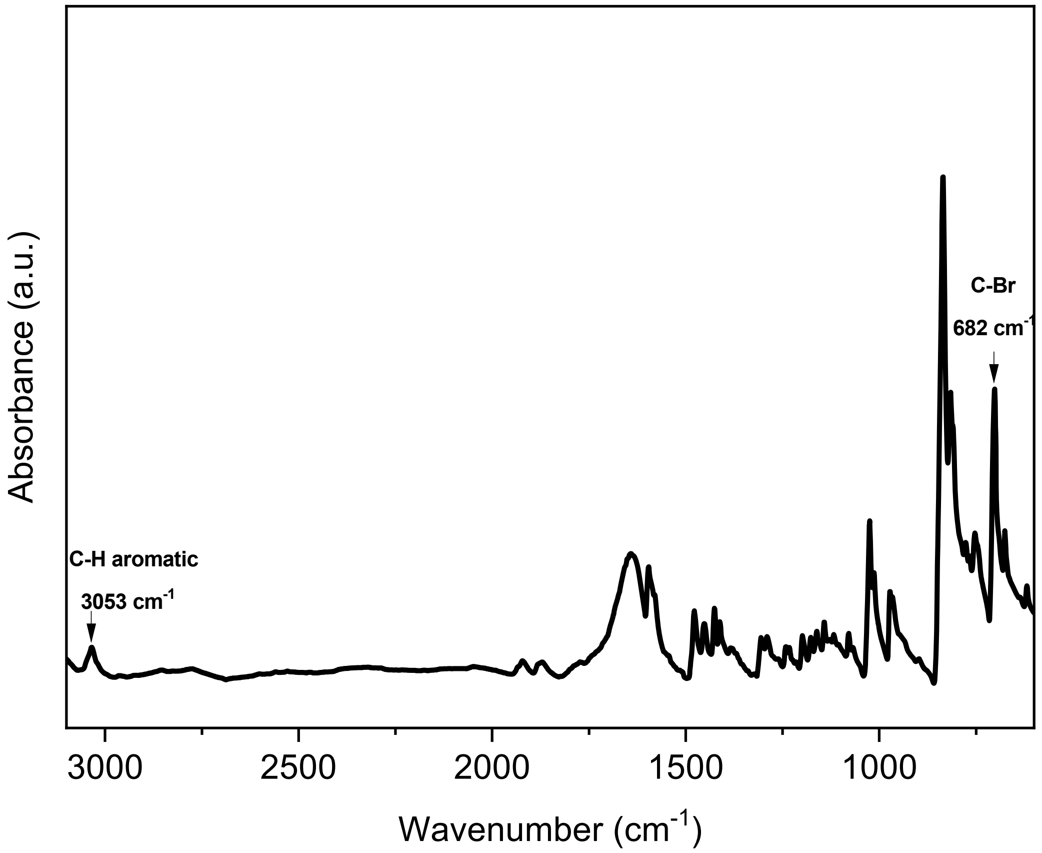

Figure S1. FT-IR spectrum of P-Br_4.

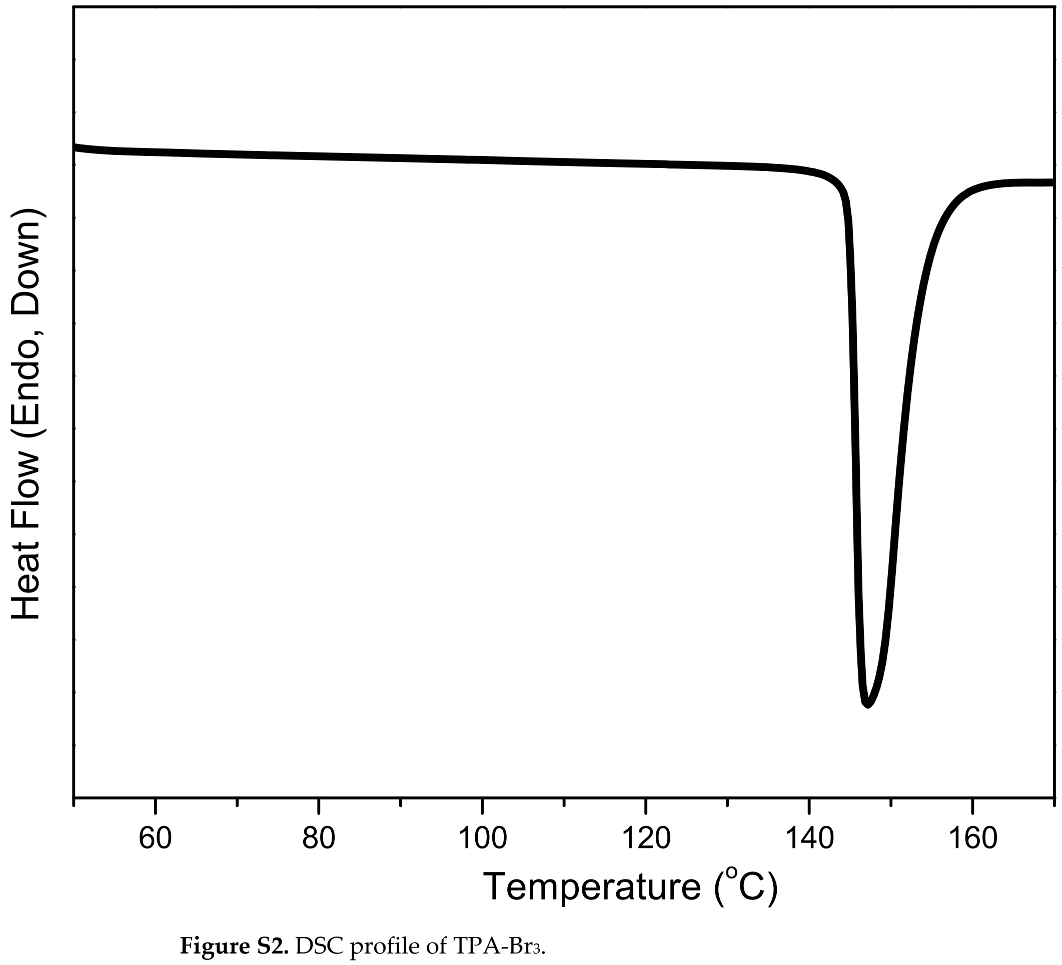

Figure S2. DSC profile of TPA-Br_3.

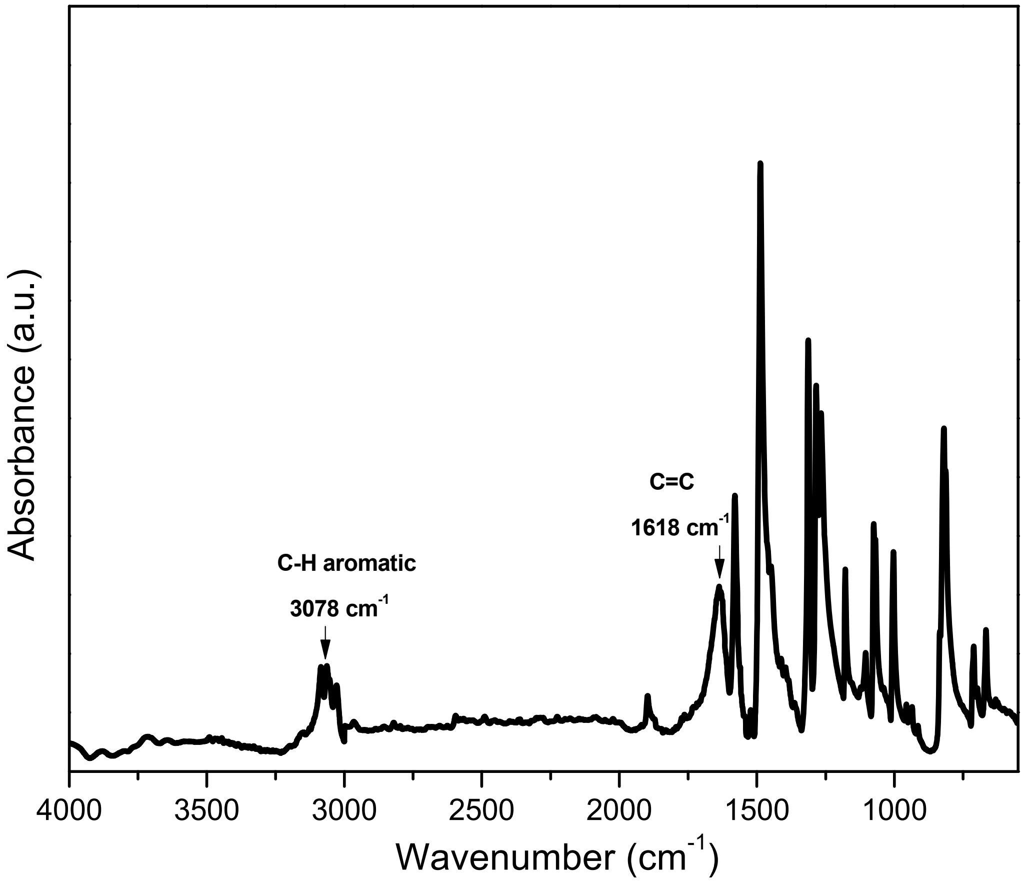

Figure S3. FT-IR spectrum of TPA-Br_3.

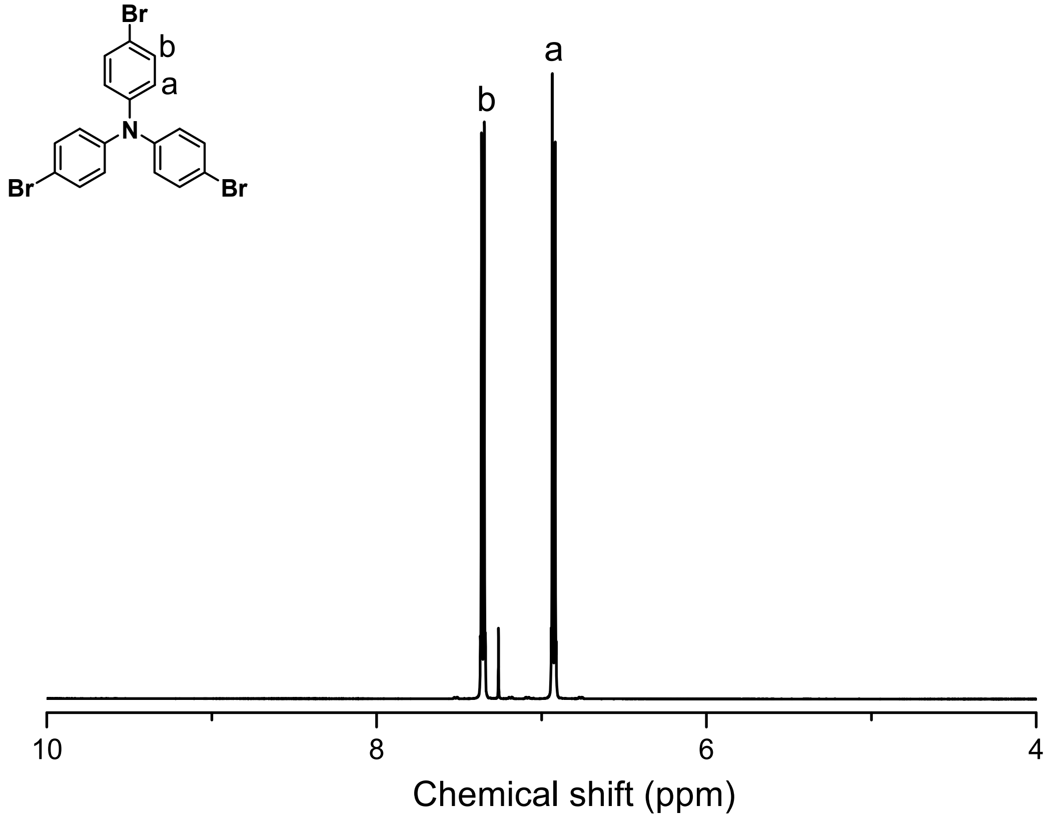

Figure S4. ^{1}H NMR spectrum of TPA-Br_3.

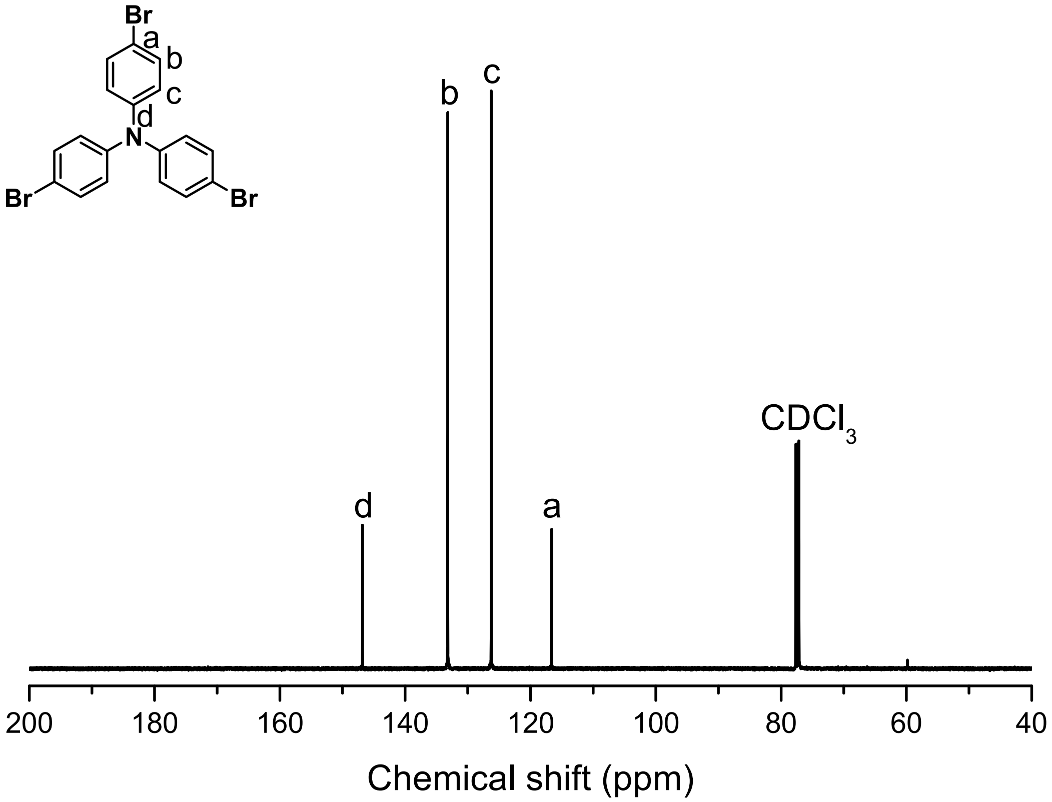

Figure S5. ^{13}C NMR spectrum of TPA-Br_3.

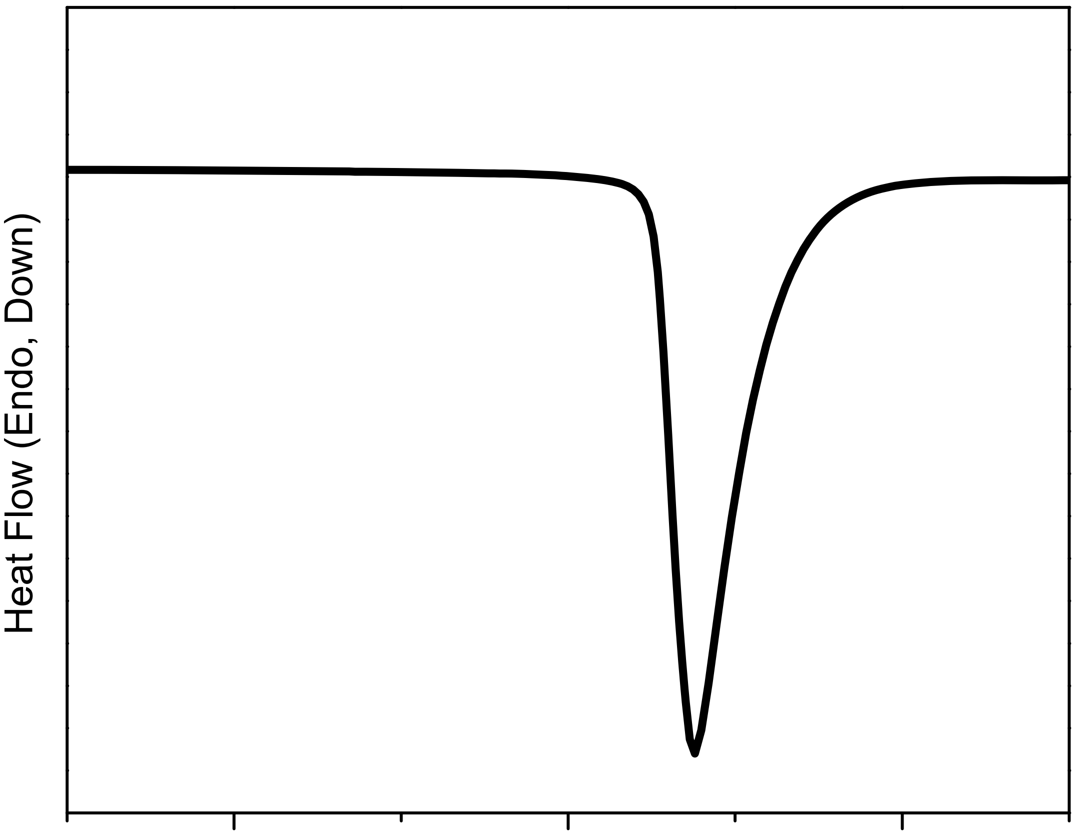

Figure S6. DSC profile of F-Br_2.

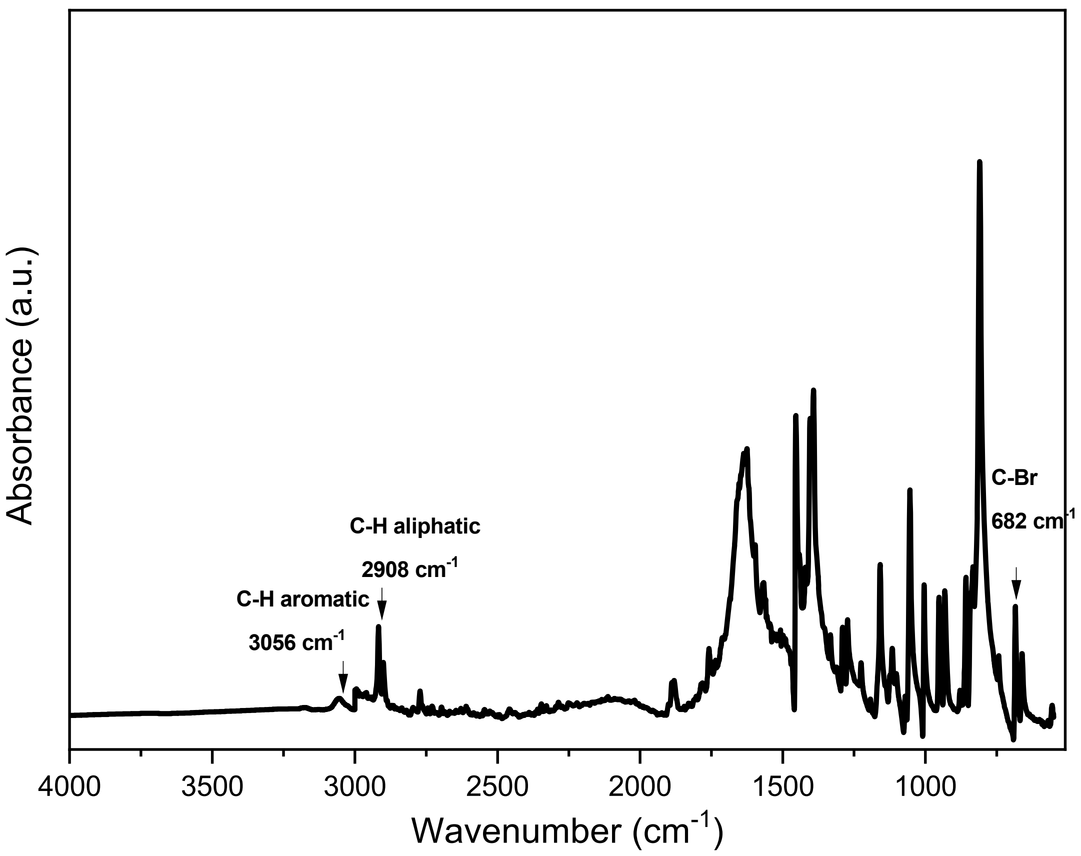

Figure S7. FT-IR spectrum of F-Br_2.

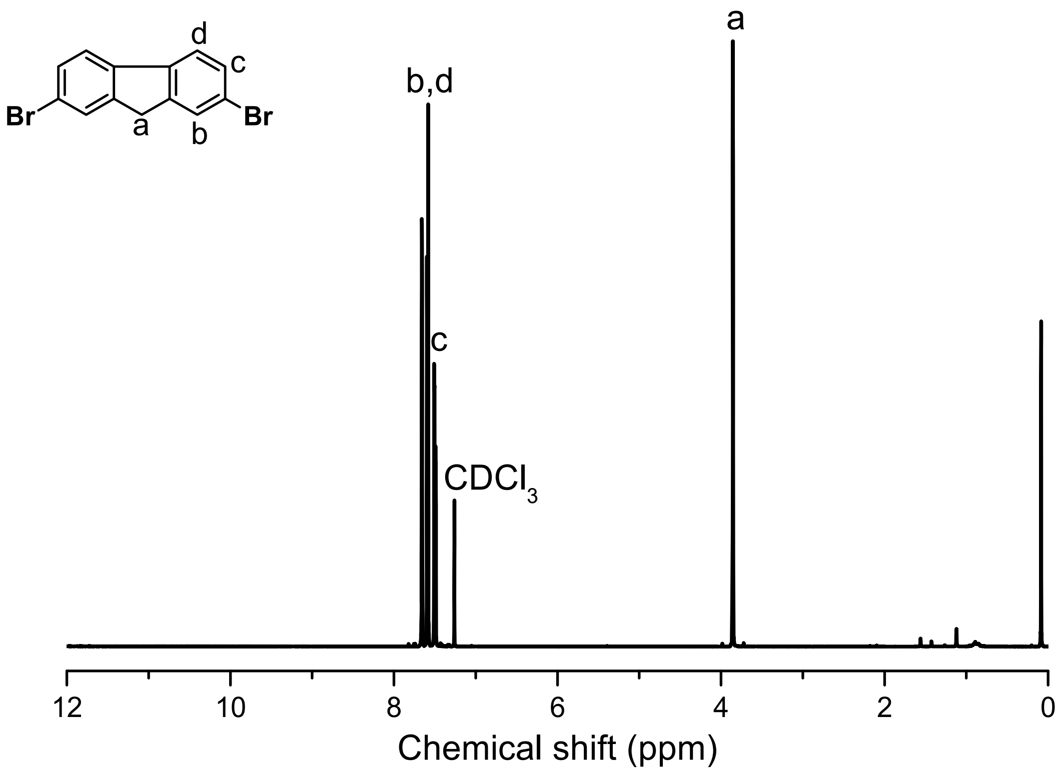

Figure S8. ^{1}H NMR spectrum of F-Br_2.

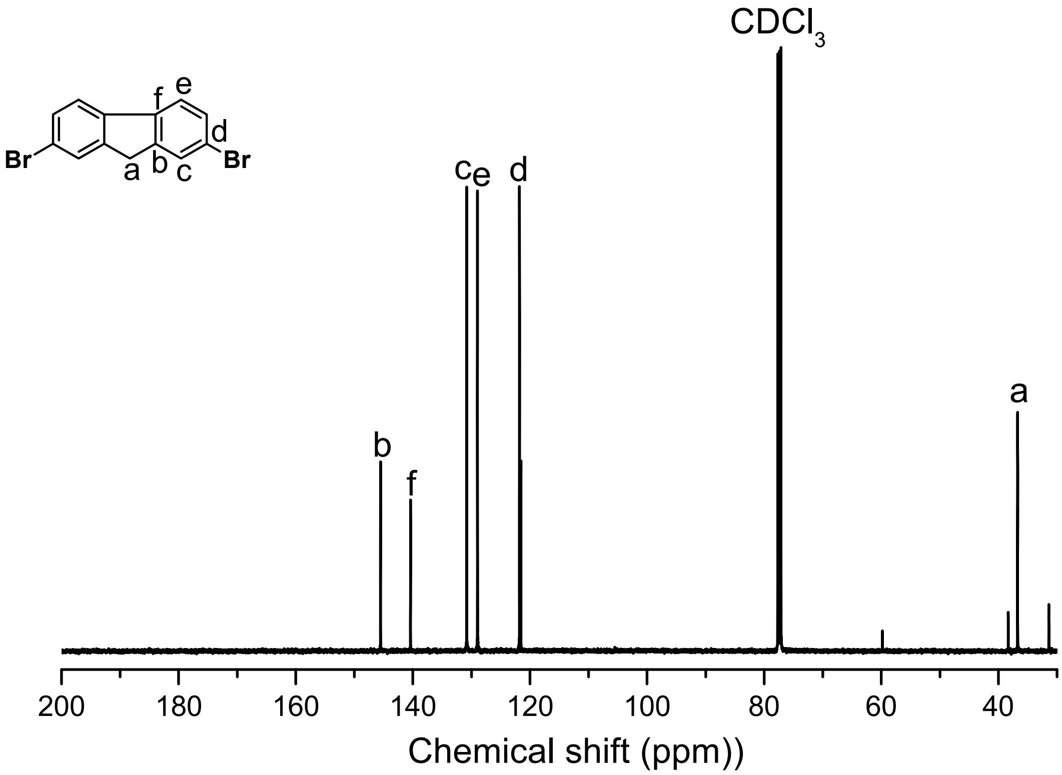

Figure S9. ^{13}C NMR spectrum of F-Br_2.

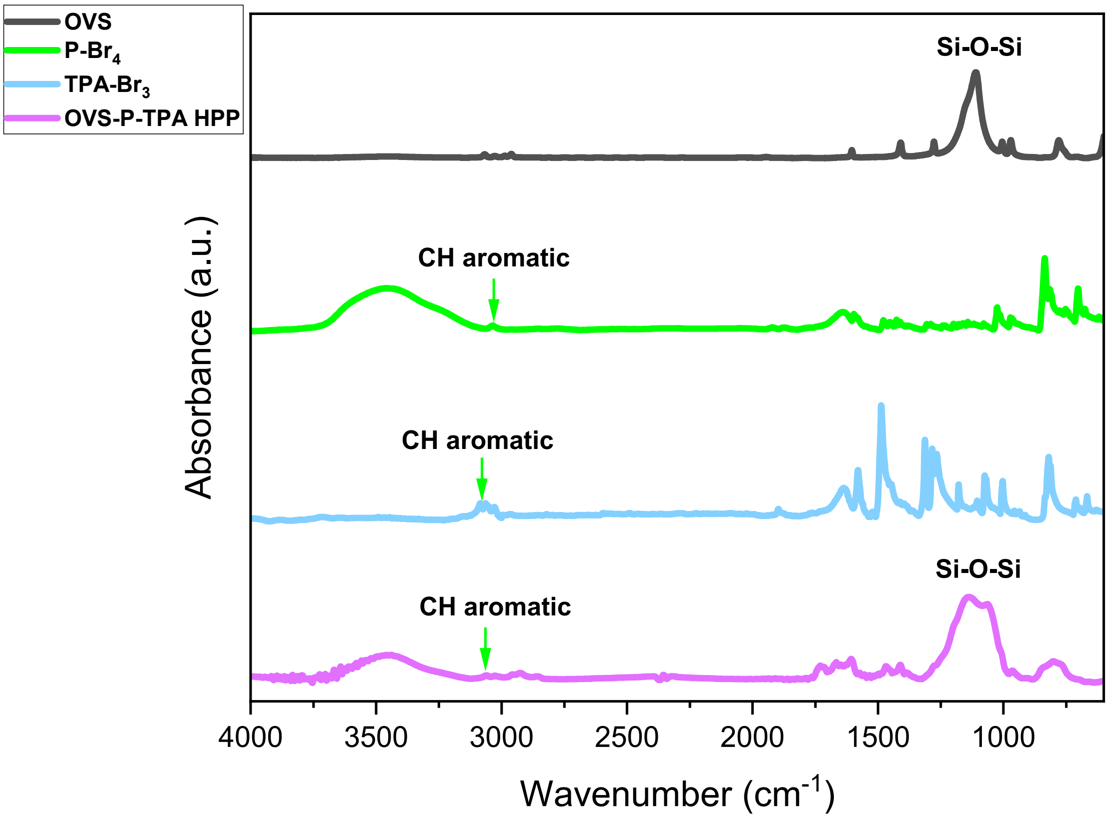

Figure S10. FTIR analyses of OVS, P-Br_4, TPA-Br_3 and OVS-P-TPA HPP.

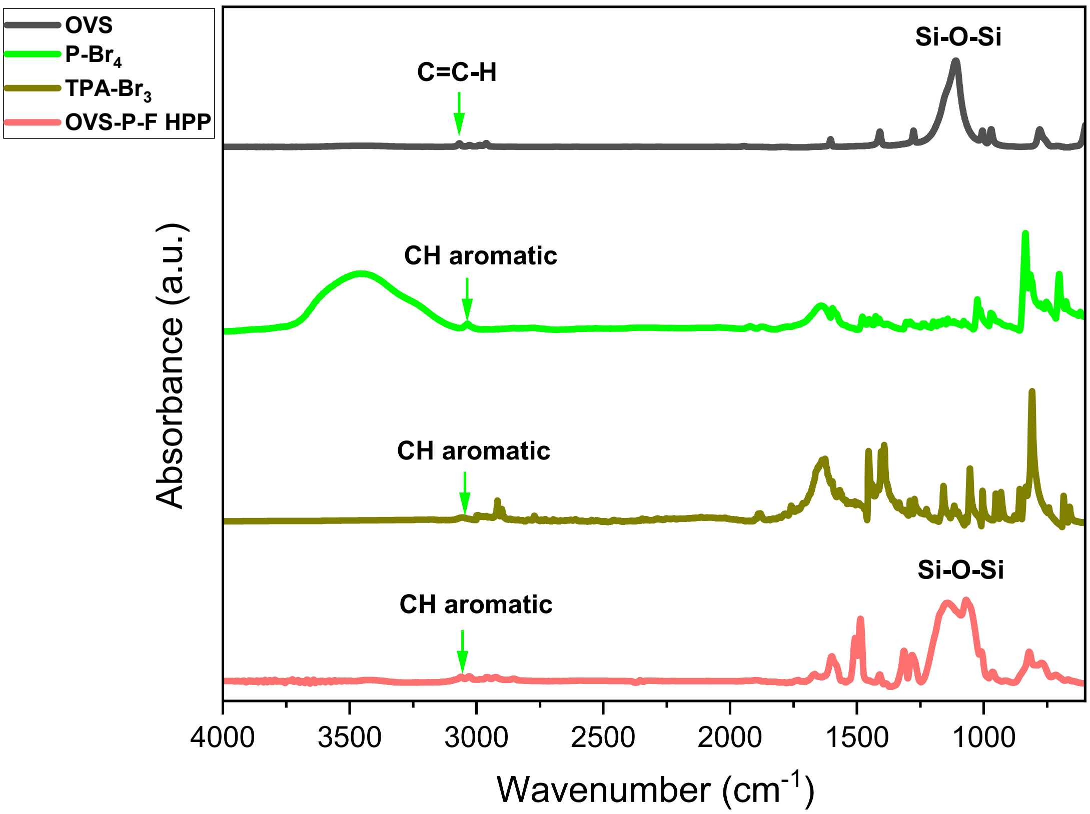

Figure S11. FTIR analyses of OVS, P-Br4, F-Br2 and OVS-P-F HPP.

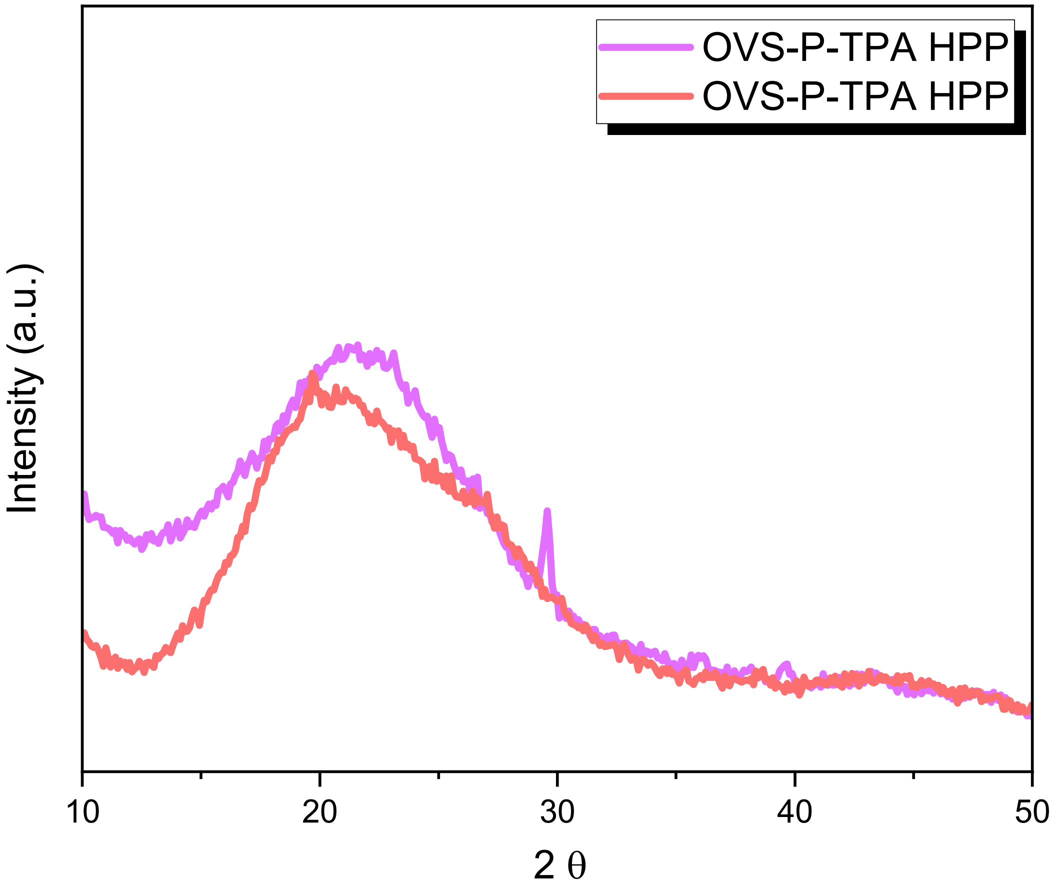

Figure S12. XRD patterns of OVS-P-TPA and OVS-P-F HPPs.
